# Biofuels 2020: Biorefineries based on lignocellulosic materials

**DOI:** 10.1111/1751-7915.12387

**Published:** 2016-07-29

**Authors:** Miguel Valdivia, Jose Luis Galan, Joaquina Laffarga, Juan‐Luis Ramos

**Affiliations:** ^1^ Biotechnology DepartmentAbengoa ResearchCalle Energía Solar n°141014SevillaSpain; ^2^ Department of Business Administration and MarketingUniversity of SevilleSevillaSpain; ^3^ Department of Financial Economics and AccountingUniversity of SevilleSevillaSpain

## Abstract

The production of liquid biofuels to blend with gasoline is of worldwide importance to secure the energy supply while reducing the use of fossil fuels, supporting the development of rural technology with knowledge‐based jobs and mitigating greenhouse gas emissions. Today, engineering for plant construction is accessible and new processes using agricultural residues and municipal solid wastes have reached a good degree of maturity and high conversion yields (almost 90% of polysaccharides are converted into monosaccharides ready for fermentation). For the complete success of the 2G technology, it is still necessary to overcome a number of limitations that prevent a first‐of‐a‐kind plant from operating at nominal capacity. We also claim that the triumph of 2G technology requires the development of favourable logistics to guarantee biomass supply and make all actors (farmers, investors, industrial entrepreneurs, government, others) aware that success relies on agreement advances. The growth of ethanol production for 2020 seems to be secured with a number of 2G plants, but public/private investments are still necessary to enable 2G technology to move on ahead from its very early stages to a more mature consolidated technology.

Biofuels produced from crops have been the driving force in renewable energies. In the first decade of the 21st century, there was a major focus on the debate of food versus fuel. Reports made by national and international agencies, such as OECD (OECD, 2008), FAO, EU and others concluded that the food commodity prices were being impacted by consumption for the production of biofuels. Other slightly later reports (Mohr and Raman, 2013) studied the sustainability of 1G ethanol production and the implications of alternative feedstocks. Influenced by the global debate, policies were implemented to promote the production of liquid biofuels from feedstocks not used for human consumption, and give rise to what is called, second‐generation biofuels (2G). Lignocellulosic materials, from herbaceous crops, hardwood and softwood, are the main feedstocks used for the production of liquid biofuels, particularly ethanol.

The main drivers behind a push towards both 1G and 2G biofuel production are (i) energy supply security and reduction in dependency on oil imports, (ii) support for rural areas through technology deployment and creation of knowledge‐based jobs and (iii) mitigation of the greenhouse gas emission (GHG), and the reduction in emissions of particulate matter that are toxic for the environment, animals and humans —promoting a low‐carbon and sustainable economy.

Over the last decade, research in the 2G market has been searching for a significant breakthrough that will lead to it being cost competitive with first‐generation biofuels. Unfortunately, the development of the lignocellulosic ethanol market has been slower than expected due to a perception of high technological risk, intensive capital costs and the low oil prices that result in poor economics for the biorefineries (Stephen *et al*., 2012). An intensive growth period (2014–2020) has been forecast and production capacity is expected to reach 2220 Million litres by 2020, from a capacity of 750 Million litres in 2014 (United Nations, 2016). Other organizations are not as optimistic but also estimate growth, for example, the OECD/Food and Agriculture Organization of the United Nations (2015) estimate a total capacity of 1703 Million litres for second‐generation ethanol by 2024, mainly in USA and Europe (UNCTAD, 2015).

In spite of discrepancies in the forecast figures for 2G ethanol, the general feeling is clear that there will be very significant growth over the next decade. Some studies have estimated the value that can be generated by the lignocellulosic industry, for example (Hertel *et al*., 2015), give a value to second‐generation industry of $64 billion under baseline conditions. Apart from the direct evolution of the lignocellulosic industry, there are other external factors that have a clear impact on the future of 2G production, in particular oil prices; for lignocellulosic biofuels to be cost competitive, an oil price in the range $70–85/barrel is required (Sims *et al*., 2009).

A focused strategy to battle climate change through regulation will strengthen the stance of alternative technologies and secure the second‐generation biofuel industry. In conjunction with climate change mitigation strategies, new technologies also require a boost to allow the development of new products. Some steps have been made globally with this regard and the USA is currently the most advanced market owing to a stable legal framework. The EU is a step behind due to social concerns and the lack of a common strategy in EU‐28. Low carbon fuel legislations in France, Italy and the UK include ethanol tariffs and antidumping penalties as barriers to biofuel production.

Analysis of the policies in the USA revealed several drivers that favour 2G ethanol. These policies were developed under the Energy Policy Act of 2005 and were published as the Renewable Fuels Standard (RFS), which was later updated by the Energy Independence and Security Act of 2007. The RFS assures that the transportation fuel sold in the USA contains a minimum volume of renewable fuel. The RFS objective (Fig. 1) is to increase the biofuel blend up to 36 billion gallons (Bgal) by 2022 from 9 Bgal in 2008.

**Figure 1:**
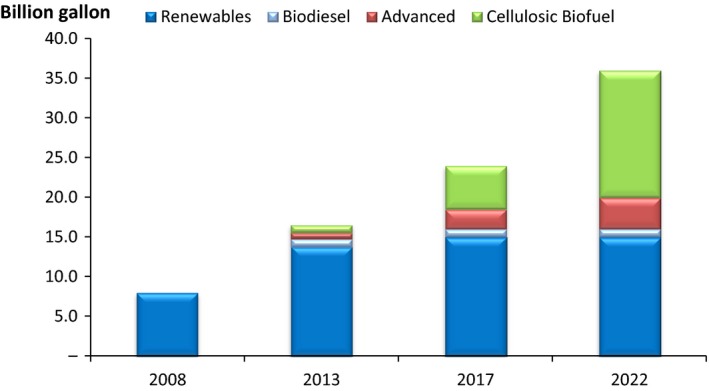
Volumes target for renewable fuel for the USA. *Source*: US Environmental Protection Agency (EPA). https://www.epa.gov/renewable-fuel-standard-program/program-overview-renewable-fuel-standard-program

To assure these targets are on track, each year the US Environmental Protection Agency (EPA) publishes the amount of biofuels that the blenders have to include in their gasoline, the Renewable Volume Obligation, is controlled by assigning identification numbers, that is, renewable identification number (RIN). The last update was published by the EPA on December 15, 2015 and the requirements are shown in Table 1. Of note, the volumes required were significantly reduced from those published in the clean air act of 2007, this is due to a delay in the deployment of the first commercial lignocellulosic bioethanol plants.

**Table 1:** Update of biofuels volume requirements for 2014–2017 according to EPA

Volume requirements	2014	2015	2016	2017
Cellulosic biofuel (million gallons)	33	123	230	n/a
Biomass‐based diesel (billion gallons)	1.63	1.73	1.90	2.00
Advanced biofuel (billion gallons)	2.67	2.88	3.61	n/a
Renewable fuel (billion gallons)	16.28	16.93	18.11	n/a

n/a: not available.

The RINs are assigned to the production facilities and are traded in a public market. The blender buys RINs at the same time as the ethanol is purchased or it goes to the market to buy the RINs and reach the requirement set by the administration. To differentiate among corn‐starch ethanol, cellulosic ethanol and other renewable fuels, several categories are defined to give an amount of blending for each type of biofuel, that is, D3 for cellulosic biofuel, ethanol and biogas. This classification is under the legislation of the RFS2 which defines different categories depending on the feedstock and the GHG reduction^1^: Renewable fuel: 20%; Advanced biofuel: 50%; Biomass based 50%; diesel: 50% and Cellulosic biofuel: 60%. Since the start of the RFS, 354 million metric tonnes of CO_2_ has been avoided (Boland and Unnasch, 2015).

In addition to the previously described blending legislations, the US includes a number of value‐generating aspects at the federal level, for example, the cellulosic waiver credit (CWC), which is a tax exemption that inversely correlates with gasoline prices. The EPA calculates the CWC each year, its price is the greater value of $0.25 or $3.00 minus the wholesale price of gasoline. For 2015 the waiver was $0.64/gal, whereas in 2016 the value may be as high as $1.33/gal and in 2017 it is likely that the waiver will be higher again due to lower gasoline prices.

It is also necessary to take the impact of the automobile industry into account. It is important that they promote the 2G ethanol industry, not only because car/truck emissions will be reduced but also because there are concerns among consumers and policy makers on the so‐called ‘blend wall’, that is, the amount of biofuel that can be blended per unit of final fuel. Legal blending in a number of countries is around 10%, this has been under discussion for several years now, and it has been proposed to increase the legal requirement up to 15%. No agreement has been reached between oil refiners, vehicle producers and the biofuel industry, and as such this move is proceeding very slowly. Experience shows that current motor vehicles could operate with E15 without any major changes.

In countries where the bioethanol industry is well developed, such as Brazil, most of the vehicles are flexi‐fuel, allowing the consumer to choose between using regular fuels or biofuel depending on prices. The Brazilian experience supports that biofuels can be used in appropriate vehicle fleets.

## What is the market situation today?

Tremendous advances have been made by the lignocellulosic industry in the last decade. In fact, at least four commercial plants have been inaugurated in the last few years: Project Liberty by the joint venture Poet‐DSM; Dupont 2G ethanol facility in Iowa; Abengoa Bioenergy Hybrid Kansas by Abengoa; and Crescentino by BetaRenewables (the only one in Europe).

All of these are at different levels of operation as they are in their current start‐up phase. In all cases, a number of issues have been encountered that have prevented full operation, and this can be expected for the use of first‐of‐its‐kind technology. The good news is that the owners expect them to be in regular operation by 2017; success in this set of facilities is crucial for the further commercial deployment of the industry.

In the US, government support for the 2G technology has been significant, but is probably still not sufficient. To date, the government has allocated large amounts of funding for R&D projects; in addition, some companies, such as Abengoa, POET‐DSM and IneosBIo have received funding for commercial facility construction. There is no doubt that this support helped to advance the state of the art for this technology, however, further support is needed to bring the technology to maturity. This support could be provided through direct funding for commercial projects, and through support using tax credit exemptions, premiums for lignocellulosic biofuels or by increasing the legal blending limit.

Investors are another necessary arm that can assist in bringing the 2G biofuel industry to a mature level; there are a set of private investors that are willing to allocate their resources to green, sustainable and economically viable technology. To have access it will first be necessary to deploy the aforementioned commercial facilities. This will decrease the perceived technological risk for investors, and increase the number of entities, banks and private funds interested in this market.

## The lignocellulosic biofuel value chain

One of the main peculiarities of the lignocellulosic biofuels is its value chain, which starts at feedstock harvesting. The availability of enough cost‐effective biomass is one of the main challenges for the industry. It should be noted that previously many agro‐wastes were left on the ground. New machinery is needed to harvest, process, transport and store the large amounts of material that are needed to make 2G biofuels. All of these factors influence the price of feedstock and unfortunately the logistics for handling and supplying feedstock are not well developed.

Different raw materials can be used as feedstock for lignocellulosic biofuels, these include agricultural residues (corn stover, wheat straw, sugarcane straw, bagasse, etc.), forestry residues (woody biomass), municipal solid waste or energy crops planted in non‐productive areas.

The challenge is not the global amount of feedstock that is available; in fact, a number of studies estimated that in the US alone, there is more than 450 Mdryton/year could be available by 2030 (U.S. Department of Energy, 2011), this amount would have the potential to produce 67 Ggal ethanol/year. The US Department of Energy suggested that there are between 600 and 1000 million tons of terrestrial biomass that should be available at price of $60/ton at origin (the farm gate).

So the problem is not the amount of biomass but the logistics of procurement. As a consequence of the lack of a well‐defined logistical model, biomass supply represents the main cost in lignocellulosic biofuel production. It should be noted that municipal solid waste is an exception here and has a different scenario. Another major issue is the cost associated with getting the biomass to destination. Today's commercial plants have transport costs up to $75 US/ton, this makes the economics of the technology non‐viable. Efforts to optimize the supply of biomass are needed. For this two lines should be stressed, on the one hand, the biomass at origin, and on the other hand the logistical model. For the former, farmers need to be made aware of the profit that could be derived from the sale of biomass for added value processes. Industry experience, both in the USA and Europe, demonstrated that farmers need to be educated in the benefits that they will obtain from the deployment of this industry. The industry must provide the relevant key information to farmers in the regions where a 2G facility is going to be constructed to create the proper atmosphere. The extra income for rural areas will increase the profitability of traditional farming.

The possibility of utilizing marginal land for the growth of biomass to be used as feedstock for biofuels is a major advantage of the lignocellulosic biofuel industry. A large amount of work has been aimed at the development of viable energy crops; these kind crops are easily adapted to the environment and ground conditions. Moreover, these viable energy crops increase two critical factors; the energy density per hectare (GJ/ha), this is, the amount of energy that can grow per hectare, and the potential biofuel yield (Gal/ton), this is, the maximum theoretical amount of biofuel that can be obtained per unit of biomass (Somerville *et al*., 2010).

One of the main challenges that any commercial plant faces in obtaining financial backing is the assurance of long‐term feedstock supply. To have a bankable project, it is necessary to have sufficient feedstock to assure the economic return of the project. This kind of contract is new for farmers and to biomass suppliers who usually work on an annual basis. Creation of agricultural associations will be an important step on the path to reaching these agreements. As mentioned above, the use of energy crops with reliable long‐term base production will also decrease the risks in supply.

Signing long‐term supply agreements is quite a challenge, particularly in Europe, where the number of participants is multiplied. For example, to assure a 300 kton per year supply of corn stover, it is necessary to reach an agreement with more than 20 000 farmers, whereas in the USA, for the same amount, it could be achieved with just 150.

Supply is not only a cost problem but it is also a location issue. Nowadays, facility location is determined by feedstock availability over a very limited distance, not more than 200 miles. To have available feedstock in a short radius, the facilities are placed in relatively remote areas, this in turn increase the rest of the production costs, such as, utilities, personnel and final product transport logistics.

One solution could be to create centralized markets or biomass reference markets that allow homogeneous supply routes, e.g. biomass pellets or chipped material in the case of woody biomass. An example could be, as described by (Lamers *et al*., 2015), an intermediate storage where preprocessing of the biomass is carried out. This would decrease logistic costs and provide higher versatility to all facilities. Standardization of biomass from different feedstocks, defining specifications regarding polysaccharide content that could be reached by more than one agriculture residue or energy crop will help to advance the 2G industry.

Another challenge is to have multifeedstock lignocellulosic biorefineries, that is, second‐generation facilities that work with heterogeneous lignocellulosic materials. If future facilities are able to process a mix of feedstock, the industry will reach the flexibility required to provide more freedom for the location of the facilities, and the number of potential facilities per region will probably increase. As such, an area to develop is the optimization of productive processes for lignocellulosic biofuels so that they operate simultaneously with different raw materials; this will make the technology less dependent on local feedstock from a given location.

## Lignocellulosic facility process description

Once the biomass is harvested, collected and transported to the facility location, the next step is to process and convert it into a liquid biofuel.

**Figure 2:**
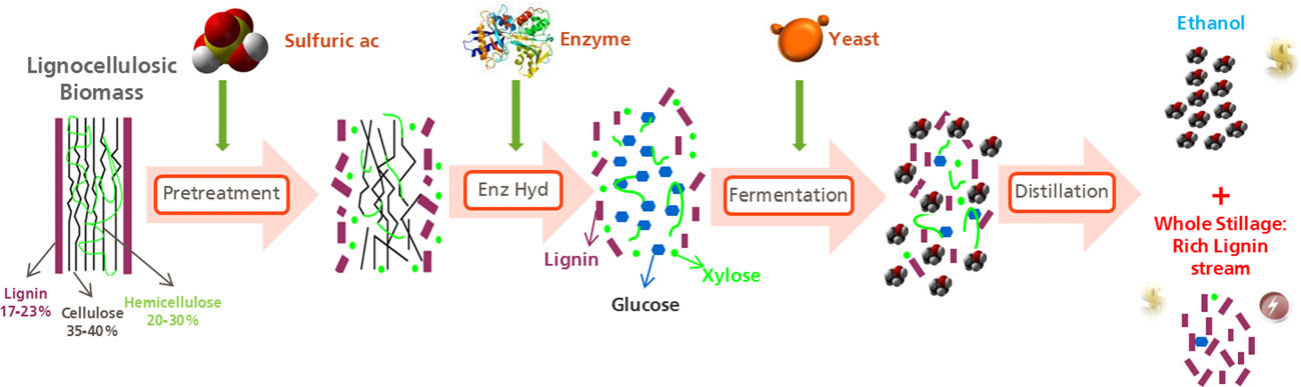
Lignocellulosic process converting the biomass into biofuels and coproducts. Process step for conversion of agricultural residues into ethanol. *Source*: Abengoa

The technology to process lignocellulosic materials is not yet fully established, with the first commercial plants finalizing commissioning and beginning start‐up in the last few years. Although many advances have been made, second‐generation biorefineries have great challenges to overcome in the next decade to become a mature and competitive technology. The procedure is mainly divided into four processes (Fig. 2): (i) pretreatment, where the cellulose and hemicellulose of the biomass are made accessible; (ii) enzymatic hydrolysis, where the biomass is converted into sugars thanks to the addition of the proper enzymes; (iii) fermentation, where the alcohol is produced from C5 and C6 monomers and, finally and (iv) distillation to produce a purified liquid fuel. In all of these areas, there is still room for technology improvement.

The aim of the storage and biomass handling areas is to receive and store harvested leftover agro‐wastes. For a 25 Mgal facility, almost 1000 tons of biomass per day is required. The storage area dimension depends on the logistic model, but it is expected to have enough capacity to maintain for at least 6 months of operation. The stored biomass must meet at least two key specifications: moisture content and ash content. The entrance to the processing area is through the biomass handling section, where the biomass, normally stored in bales, is de‐stacked, de‐stringed and size reduced to reach the designed particle size distribution (PSD); finally, ground material is screened to remove fine particulate, which have high ash content. The output from the biomass handling goes to the biofuel process conversion area and if the facility has an integrated biomass boiler, part of the biomass will be allocated to be burned.

Biofuel conversion starts with pretreatment, which consists of an acid or alkaline soak system that saturates the feedstock with dilute strong acid or base. The soaking system also removes a significant amount of sand, which can cause severe erosive damage to the equipment. The soaked biomass is pretreated in a continuous steam explosion reactor. The pretreatment process solubilizes the hemicellulosic sugars, primarily xylose and arabinose, and greatly improves the cellulose digestibility through disintegration from lignin.

The pretreatment process is followed by a conditioning step to adjust pH, temperature and total solids content. Afterwards, the enzyme is added to the conditioned pretreated biomass slurry to reduce the viscosity in a continuous liquefaction tower to the point at which the slurry can be easily pumped to saccharification tanks (Álvarez *et al*., 2016). Sufficient residence time in the saccharification tanks is maintained for the conversion of the cellulose into monomeric glucose and hemicellulose to glucose, xylose and other sugars of lower concentration.

The saccharification is followed by simultaneous fermentation of xylose and glucose to ethanol using a genetically modified strain of brewer's yeast (*Saccharomyces cerevisiae*). Other organisms such as *Clostridium* and *Pseudomonas* can be used to produce alternative biochemicals (Ragauskas *et al*., 2014; Tolonen *et al*., 2015; Ramos *et al*., 2016; Sanford *et al*., 2016).

Another option exists in recovering the lignocellulosic sugars to be sold as raw material for biochemical processes in plants located in a radius of about 500 km. In this case, it is necessary to include solid–liquid separation steps that permit the sugar to be purified to a level that can be used in further processes.

In a standard 2G facility, ethanol is distilled and dehydrated using conventional distillation and molecular sieve steps. The semi‐solid residue from the distillation process, called whole stillage, is separated into a liquid stream referred to as thin stillage and a soil cake using a filter press; the cake is a fraction enriched in lignin. The thin stillage is concentrated to produce ~50% solid syrup through seven stages of multieffect evaporation. The cake and syrup have different options to extract their value; currently, the most common one is to burn it on‐site, or in a closely located biomass burner, or to sell it as raw material to be used in soil amendment. The raw materials can be used in soil amendment because composition allows to increase organic C content and provides porosity to substrates that facilitate seed germination and plant root development.

We have so far described the ‘core areas’ required for lignocellulosic biofuel production. However, depending on the location or on the type of project, other areas, such as a biomass boiler or wastewater treatment plant, have to be included within the facility to comply with environmental legislation or to generate the vapour needed for plant operation.

The facility cost should take into account both the capital cost needed per gallon (Capex) and the cost to operate the plant (Opex). Both are influenced by the kind of project. There are three main types of projects:

### Greenfield/standalone

This is a plant by itself. It needs a complete value chain management. Its location influences the Capex and also the logistics of biomass supply and ethanol sales. In terms of Capex, it needs the construction of the core process, biomass handling, pretreatment, enzymatic hydrolysis, fermentation and distillation; and also auxiliary operation units such as cogeneration and a waste water treatment plant; these usually significantly increase the overall cost.

### Colocation/Bolt‐on

In this concept some existing infrastructures and operations can be shared thanks to the proximity of other industries. This model requires at a minimum the construction of the process area. The value chain for feedstock procurement and logistic must be completely developed. The construction of auxiliary operations units is limited.

### Hybrid/integrated

The whole value chain is completely integrated within a 1G facility, taking advantage of the synergies in feedstock supply and product logistics. The construction of auxiliary operation units is not necessary.

The aim of the two last configurations is to decrease the cost of the auxiliary or complementary operation units and focus the efforts on reducing the cost of the technological core areas.

For example, for a green field project the cogeneration area may represent up to 30–35% of the total equipment costs, as such choosing a location where the energy instead of being produced on site, could be bought at regular prices from close industries or facilities represents a significant advantage. Some other issues, such as cake and syrup destination need, however, to be solved.

After feedstock availability, reducing the investment cost through the reduction in the auxiliary equipment is the second step towards making a commercially viable project. The challenge, to the industry, is to be as flexible as possible to increase the potential locations where to place viable technical and economical projects.

There are a range of alternatives, outside improving the logistic model, where the industry is working to reduce operational capital costs. These include enzyme cost reduction by improving activity, valorization of the lignin contained in the raw material, increasing the pretreatment efficiency or improving the yeast production organism.

## Cost structure

**Figure 3:**
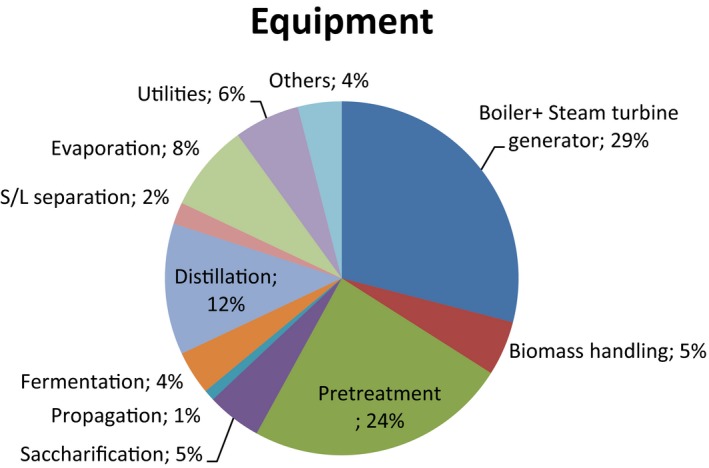
Equipment cost area distribution of a general lignocellulosic greenfield facility. Pretreatment and Boiler are the most cost‐intensive areas. *Source*: Abengoa

The location of a plant will definitively influence the overall facility layout. Investment costs will differ significantly depending on the configuration required. For a green field stand‐alone project, a non‐core area such as cogeneration increases the initial investment needed by more than 30% (Fig. 3). Absence of synergies with external utilities concomitantly results in an increase in storage equipment and logistical costs.

A colocated plant may require less than 50% in capital investment than a stand‐alone plant. Within this strategy, Poet‐DSM has colocated a 2G facility within a 1G plant. Our estimates are that investment cost ranges between $10 and 14/gal, for a plant with a nominal plate of 25 Mgal according to its location.

## Biomass handling and pretreatment

As described above, the first step in a lignocellulosic facility is to prepare the biomass for processing. This is critical because it influences all of the downstream processing. We can distinguish two main steps, one where biomass is just preprocessed to obtain the designed PSD and, in the subsequent step, the biomass is pretreated to make hemicellulose and cellulose accessible for cellulases.

For the biomass handling area, the main objective is an appropriate delivery of milled feedstock to the pretreatment system with a consistent quality that meets the required specifications in terms of particle size and total ash content. As mentioned in the feedstock section, the logistics model is critical to obtain a homogenous stream at the beginning of the process. No special innovations have been made in this area, but some experts are starting to develop different methodologies that will improve the facility performance and also the integrated logistic model.

Once the biomass has gone through the handling system, the next step is the pretreatment that will release the oligomers to be transformed into sugars. This is a critical step in lignocellulosic biofuel production. Each market player has developed their own technology as the feedstock defines the pretreatment technology and conditions. One of the main hurdles that the industry faces is that the pretreatment needs to be optimized for each raw material, limiting the flexibility of the plant to process different feedstocks. That is why more versatile pretreatments are required so as the process becomes less raw material dependent.

Pretreatment is the ‘core’ area that is most capital intensive, requiring an investment that may represent between 30% and 50% of the total equipment cost. One of the main challenges is the intrinsic recalcitrance of the lignocellulosic biomass which results in lower biomass to sugar yields and therefore in the higher pretreatment costs (Stephen *et al*., 2012).

A number of technologies are available today for the pretreatment of lignocellulose, including, chemical, physical and biological processes. Some of these technologies have already been commercialized and are well known, whereas others are still at lab scale.

The most relevant commercial technologies are given in Table 2:

**Table 2:** Pretreatment technologies

Process	Company	Characteristic
Steam explosion	Beta Renewables	Low xylose yield High enzyme loading
Single‐stage dilute acid	Abengoa	High xylose yield Moderate enzyme loading
Two‐stage dilute acid	Poet‐DSM	High xylose yield Low enzyme loading
Ammonia & Steam	Dupont	Require high enzyme loading

Each industrial player has developed its own pretreatment technology.

The 2G commercial technologies are protected by a number of patents that guard the technology while the economic viability of the projects are improved. This is why different pretreatment have been considered. The different strategies result in a series of advantages/disadvantages that are enumerated in Table 2.

Steam explosion is a well‐known advanced technology which consists of heating the biomass in water under pressure followed by a sudden decompression of the reaction vessel. As a result of the violent decompression, the structure of lignocellulose is disrupted and the fibres are opened up, leaving sugar polymers more accessible to the subsequent enzymatic hydrolysis (Stelte, 2013). As no chemicals (other than water) are used, equipment corrosion is minimal and requirements for the reactor metallurgy are less demanding. Also, the level of release of chemicals that may act as inhibitors in the saccharification or fermentation steps is very low. The main drawbacks of steam explosion are related to the mildness of the process which limits the effectiveness of the pretreatment and demands the use of very high enzyme loads in the saccharification step. Also, steam explosion is not effective on high lignin content softwood samples, and the effectiveness with hardwood is also limited (Brownell *et al*., 1986; Yang and Wyman, 2008).

Another option is the use of dilute acid in the pretreatment, this involves the use of dilute aqueous solutions of inorganic acid (HCl, H_2_SO_4_) combined with temperature. This pretreatment results in good depolymerization and release of hemicellulose and cellulose. Compared to steam explosion, dilute acid pretreatment is more efficient for woody samples. As acidic conditions allow partial depolymerization of hemicellulose and cellulose, the enzyme loading required is lower compared to simple steam explosion. However, this kind of pretreatment requires high capital investment due to the special reactor metallurgy; operational costs are also higher. It is well known that *aqueous ammonia* treatment allows biomass delignification without a significant degradation of sugars. However, the effectiveness of this pretreatment with some feedstock, such as woody biomass residues is rather limited (Yang and Wyman, 2008).

Commercial pretreatments for corn stover, wheat straw and sugar cane straw are currently being optimized, however, one of the main challenges that the sector must overcome in the coming years is to enhance process versatility to be able to deal with more than one raw material at a time. In terms of operational cost, pretreatment consumes chemicals and steam, this can represent up to 20–25% of the total operational costs.

Lignin removal is another key step in the development of the biofuel industry; in the current methodology, lignin is maintained until the distillation phase. However, there are several pretreatment technologies in development that try to separate the components of the biomass in different streams, one of the most promising is the use of ionic liquids (ILs) which are able to dissolve lignocellulose under mild conditions, resulting in more accessible cellulose and recovery of lignin in the raw material. Nevertheless, there are still challenges to the industrial deployment of this technology, including high cost and regeneration of ILs (Tadesse and Luque, 2011).

Searching for other alternatives may reduce costs and increase the possibility of using lignin in new ways that are not currently used to add value. Further discussion on the lignin issue follows below.

In the first commercial plants, one of the main bottlenecks in the commissioning and start‐up phase was achieving suitable performance in the pretreatment area. Problems such as blockage due to non‐defined PSD, not achieving the pretreated biomass specifications and not being able to reach high enough downstream have all been major hiccups.

The main challenges in pretreatment are as follows: development of a versatile technology that can reach appropriated levels of pretreated biomass material independently of the raw material used; generation of combination plants that can more easily process under milder conditions to allow optimization both economically and environmentally; to optimize pretreatment processes using less corrosive chemicals making construction materials cheaper and as consequence reduce the initial investment (in particular, steel alloys resistant to acid or base).

## Enzymatic hydrolysis

After the material is conditioned in the pretreatment area, the next step, the enzymatic hydrolysis or saccharification is one of the most critical factors in lignocellulosic biofuel production, and represents one of the main technology development areas. Enzymatic hydrolysis represents the second main operational cost, after the biomass production; in 2G it is ~25–30% of the operational costs, whereas in 1G it is below 3%.

Why this difference? The answer is the enzymatic cocktail needed for 2G. These cocktails have a combination of a wide range of activities and properties, such as cellulases, hemicellulases and β‐glucosidases that convert the polysaccharides into C6 and C5 sugars (Álvarez *et al*., 2016). Most of the enzymes used in the conversion to sugars are of microbial origin, but have been ‘hitched’ to other than their native host, that is, they area engineered. Today only about three companies have commercialized this kind of cocktail: Novozymes, Dupont and Abengoa.

In the market there is a consensus that the final enzyme cost contribution should be stabilized around $0.4/gal. Nowadays, this cost contribution is achieved at least at a demo scale by the enzymatic cocktail developed by Abengoa, although the performance on a regular basis at commercial scale is still a pending issue. Reported commercial data indicate that the three commercial cocktails may operate within the same range. Reducing the enzymatic cocktail cost contribution is critical for the viability of the 2G technology because the cost of enzymes can be up to 30%.

How can these cocktails be improved? There are several possibilities, (i) reduce the enzyme loading by improving the enzyme activity through genetic engineering using high‐performance mutagenesis strategies coupled with massive tracking systems, (ii) reduce the cost of protein through better production methodologies and (iii) increase the overall hydrolysis yield by adapting the enzyme cocktail performance to the process conditions. For the last one the exploration of extreme environments and the use of metagenomic techniques that allow the identification of new enzymes that are more efficient is a critical focus (Elleuche *et al*., 2014). The most relevant aspect is the use of thermophilic enzymes because the increase in temperature prevents hydrolysates from being contaminated and, therefore, the loss of raw material; this increases the overall performance of the process.

Another factor is that the total solid ratios at which the technology can work is relatively low compared with 1G, this is due to the lower yield per tonne of raw material introduced and the longer residence times needed. In 2G the typical solid ratio is 20% versus 33% for 1G. Although the enzymes would work even better at lower solid concentration, this is the minimum ratio at which ethanol concentrations are reached. One of the main improvements that can be made is to increase the performance of the enzyme cocktail at higher total solid, or at least reaching optimum performance at current levels.

As noted above, the 2G enzyme cocktail seems to be a niche market with very few players. A significant increase in the volume of 2G enzyme market is required to provide producers with the equity to optimize their cost structure. Enzyme producers need to be involved in the industrial development process if they want 2G facilities to achieve their nominal capacity. If the market is converted into an oligopoly with few competitors gaining most of the value from the 2G technology, the development of the industry globally will become difficult. Enzyme developers need to be involved in the industry growth, participating in different ways with not only the industrial developers but also with other stakeholders.

## Fermentation

After the enzymatic hydrolysis, a sugar stream is obtained that differs from the first‐generation sugars in that the amount of C5 sugars represents almost 30% of total sugars, C5 sugars are released from the hemicellulose with xylose. The current fermentation technology produces ethanol, although other alternative alcohols or bioproducts can be synthesized, e.g., alkanes or long‐chain alcohols, butanol, jet‐fuels, etc.

The fermentation of glucose and xylose in the same reactor can be considered a well‐developed technology, with conversion yields of more than 95%. Very efficient yeasts have been designed and optimized to ferment xylose and glucose simultaneously. While this is true with herbaceous lignocellulosic material, when the raw material comes from wood, a series of inhibitors are generated in the pretreatment that make the fermentation processes less efficient (Heer and Sauer, 2008; Tomás‐Pejó and Olsson, 2015). Another very important aspect is to shorten the fermentation time; this could lead to a reduction in the number of fermenters per plant with a consequent saving in the initial investment. In addition, it should be possible to work at different pHs or temperatures, to improve the propagation phase.

In this area, unlike at the enzyme market, there is no risk of an oligopoly which could cap the market and there is still room for improvement in terms of operational conditions to decrease the overall costs.

The processing of C5 is a must. It is not economically viable to process lignocellulosic biomass without getting any value from the hemicellulose or the lignin contained in the raw material. Several production platforms, using bacteria or yeast, are under investigation but none of them have as yet reached high enough productivity.

Some experts predict there will one day be a lignocellulosic sugar market, but today's oil prices make it non‐competitive, except for in some niche cases where the upstream product is produced outside of the sugar production facility. This is the reason why so many joint ventures and co‐ops have arisen in this market in recent years.

The industry must look for win–win agreements. This will include, on one hand, the 2G technologists or the companies that have the necessary technology to reach affordable lignocellulosic sugars and, on the other hand, the final product companies (chemical companies) that can benefit from green sugars and alcohols.

Clearly the final market needs will accelerate the product development based on the specification requirements and continuous collaboration between technologists and final clients.

## Distillation, solid and liquid separation and evaporation

The final step in reaching the product is the distillation of the stream, which comes out from the fermenters. The distillation of ethanol, if the stream is produced on specification, is an area that does not present major issues. It represents between 15% and 20% of total equipment costs. A critical factor in this step is the use of the stillage produced during the distillation. To have an economically feasible technology, the valorization of this stream product is mandatory. The stillage has a high concentration of lignin, 20–30% depending on raw material source. At present, in commercial plants, the stillage, following separation of the water content, is sent to the cogeneration area to be burned to provide power vapour to the 2G process. This waste is currently burned in cogeneration plants where the value of the generated energy is $5–10 US/ton. To increase the profitability of the plants of lignocellulosic material, it is necessary to add value to the stillage. In the configurations where there is not a cogeneration area within the plant or nearby, the lignin stream is used for irrigation in areas close to the facility. The main problem is the large volume produced. For example, a 25 Mgal plant produces around 300 000 ton/day of stillage. The other option is to send it to a wastewater treatment plant, if the configuration has it, or send it to a disposal agency; both of which represent a significant extra cost for the plant (Ramos *et al*., 2016).

So, to increase the value obtained for this coproduct, one of the main challenges of this industry is to valorize the stillage as was done with fibre in the first‐generation industry. In the process of ethanol generation from corn or other cereal grains, the economy of the process rests not only in the sale of alcohol but also in the grain residue that is left, which is known as DGG and is used as animal feed (Patrik R. Lennartsson, 2014).

As has been stated, the stillage contains up to 30% lignin within its composition coming from the lignocellulosic biomass. Lignin is a natural polymer with excellent properties that can be used in the chemical industry. A number of reviews on lignin valorization are available (Ragauskas *et al*., 2014), but there are few success cases that have been implemented. A difference that 2G lignin has compared to commercial preparations, principally those which come from the pulp and paper industry, is the absence of sulfur; this is a great advantage for its use in some chemical applications such as resins or composites. Most 2G technologists are struggling with themselves on how to increase lignin value. The current proposal is to extract lignin from this waste to be used in the synthesis of new resins to replace those based on chemical compounds derived from the petrochemical industry.

Once the technology has been fully developed and the main challenges resolved the question is as follows: Would the market be ready for this new product?

## Concluding remarks

Production of liquid biofuels from grains in the so‐called 1G generation is a mature technology that seems to have plateaus in technology and production capacity. An increase in the production of ethanol is foreseen to be the result of the conversion of agricultural wastes and municipal solid residues into ethanol using the so‐called 2G technology. A number of 2G plants have been built, but their operability at full nominal capacity has not been attained so far due to mechanical hurdles. The identification of these limitations, their potential solutions and combined public/private investment will be crucial to enable 2G technology to move on ahead from its very early stages to a more mature consolidated technology. International expert agencies believe that an estimate of 15 2G plants will be built by 2024. In our view, we also foresee that ethanol will not be the only product to be derived from polysaccharides originated from agricultural residues and wastes, but also another more potent liquid biofuel, such as butanol, is making symbiotic biofuels part of this new market.

## Conflict of interest

None declared.

## Note

1 Compared to a 2005 petroleum baseline.
